# The atlas of supine single port extraperitoneal access

**DOI:** 10.1590/S1677-5538.IBJU.2024.0400

**Published:** 2024-08-15

**Authors:** Luca Lambertini, Matteo Pacini, Luca Morgantini, Jhon Smith, Juan Ramon Torres-Anguiano, Simone Crivellaro

**Affiliations:** 1 University of Illinois at Chicago Department of Urology Chicago Illinois USA Department of Urology, University of Illinois at Chicago, Chicago, Illinois, USA; 2 University of Florence Careggi Hospital Department of Experimental and Clinical Medicine Florence Italy Department of Experimental and Clinical Medicine, University of Florence - Unit of Oncologic Minimally-Invasive Urology and Andrology, Careggi Hospital, Florence, Italy; 3 University of Pisa Department of Translational Research and New Technologies in Medicine and Surgery Pisa Italy Urology Unit, Department of Translational Research and New Technologies in Medicine and Surgery, University of Pisa, Pisa, Italy

## Abstract

**Introduction:**

The introduction of Single-Port (SP) platform opened the field to new surgical options, allowing to perform major urological robot-assisted procedures extraperitoneally and with a supine patient positioning (1–3). Nevertheless, a comprehensive description of different supine access options is still lacking (4–6). In this light, we provided a step-by-step guide of SP extraperitoneal supine access options also exploring preliminary surgical outcomes.

**Materials and methods:**

Transvesical access was performed by a transversal incision 3cm above the pubic bone, after the anterior abdominal sheet incision, the bladder was insufflated with a flexible cystoscope and the detrusor muscle was incised at the level of the bladder dome. Similarly, the extraperitoneal access was carried out with a 4cm incision above the pubic bone, once visualized the preperitoneal space the prevesical fat was gently spread. The Low Anterior Access was performed with a 3cm incision at the McBurney point, the abdominal muscles were then spread. A gentle dissection was used laterally to develop the retroperitoneal space.

**Results:**

Overall, sixteen different procedures were performed with supine extraperitoneal access on 623 consecutive patients. No intraoperative conversions occurred. The median access time was 16 (IQR 12-21), 11 (IQR 7-14) and 14 (IQR 10-18) minutes in case of transvesical, extraperitoneal and low anterior access, respectively. Notably, 81.5 % of patients were discharged on the same day with a postoperative opioid free rate of 73%.

**Conclusion:**

The Atlas provides a comprehensive step-by-step guide to successfully perform all major urological SP procedures extraperitoneally and with supine patient positioning.

**Available at:** http://www.intbrazjurol.com.br/video-section/20240400_Lambertini_et_al
